# Phase I Trial of Oral Yeast-Derived β-Glucan to Enhance Anti-GD2 Immunotherapy of Resistant High-Risk Neuroblastoma

**DOI:** 10.3390/cancers13246265

**Published:** 2021-12-14

**Authors:** Fiorella Iglesias Cardenas, Audrey Mauguen, Irene Y. Cheung, Kim Kramer, Brian H. Kushner, Govind Ragupathi, Nai-Kong V. Cheung, Shakeel Modak

**Affiliations:** 1 Department of Pediatrics, Memorial Sloan Kettering Cancer Center, New York, NY 10065, USA; iglesiaf@mskcc.org (F.I.C.); cheungi@mskcc.org (I.Y.C.); kramerk@mskcc.org (K.K.); kushnerb@mskcc.org (B.H.K.); cheungn@mskcc.org (N.-K.V.C.); 2 Department of Epidemiology-Biostatistics, Memorial Sloan Kettering Cancer Center, New York, NY 10065, USA; mauguena@mskcc.org; 3 Department of Medicine, Memorial Sloan Kettering Cancer Center, New York, NY 10065, USA; ragupatg@mskcc.org

**Keywords:** β-glucan, neuroblastoma, anti-GD2 antibody

## Abstract

**Simple Summary:**

Beta glucans, complex polysaccharides, can prime leukocyte dectin-1 and CR3-receptors and enhance anti-tumor cytotoxicity of monoclonal antibodies. In a phase I study (clinicaltrials.gov NCT00492167), we treated patients with intravenous 3F8 (fixed dose of 10 mg/m^2^/day × 10 days) and oral BG (dose-escalated from 10–200 mg/kg/day × 17 days in cohorts of 3–6 patients each). Forty-four patients completed 141 cycles of therapy with 3F8 + BG. One patient developed DLT: transient self-limiting hepatic transaminase elevation at a BG dose of 120 mg/kg/day. Overall, 1, 3, 12 and 24 evaluable patients had complete response, partial response, stable and progressive disease, respectively, at the end of treatment. Positive human anti-mouse antibody response and dectin-1 rs3901533 polymorphism were associated with better overall survival. BG dose level and serum BG levels did not correlate with response. BG lacked major toxicity and, in combination with 3F8, demonstrated anti-neuroblastoma activity against resistant disease. The recommended phase II dose was established at 40 mg/kg/day.

**Abstract:**

Beta glucans, complex polysaccharides, prime leukocyte dectin-1 and CR3-receptors and enhance anti-tumor cytotoxicity of complement-activating monoclonal antibodies. We conducted a phase I study (clinicaltrials.gov NCT00492167) to determine the safety of the combination of yeast-derived beta glucan (BG) and anti-GD2 murine monoclonal antibody 3F8 in patients with relapsed or refractory high-risk neuroblastoma. Patients received intravenous 3F8 (fixed dose of 10 mg/m^2^/day × 10 days) and oral BG (dose-escalated from 10–200 mg/kg/day × 17 days in cohorts of 3–6 patients each). Forty-four patients completed 141 cycles. One patient developed DLT: transient self-limiting hepatic transaminase elevation 5 days after starting BG (120 mg/kg/day). Overall, 1, 3, 12 and 24 evaluable patients had complete response, partial response, stable and progressive disease, respectively, at the end of treatment. Positive human anti-mouse antibody response and dectin-1 rs3901533 polymorphism were associated with better overall survival. BG dose level and serum BG levels did not correlate with response. Progression-free and overall survival at 2 years were 28% and 61%, respectively. BG lacked major toxicity. Treatment with 3F8 plus BG was associated with anti-neuroblastoma responses in patients with resistant disease. Although the maximal tolerated dose for yeast BG was not reached, considering the large volume of oral BG, we recommended 40 mg/kg/day as the phase II dose.

## 1. Introduction

Neuroblastoma (NB) is the only pediatric solid tumor for which anti GD-2 IgG monoclonal antibodies have demonstrated a significant survival benefit [1]. The chimeric anti-GD2 monoclonal antibodies (mAb) dinutuximab [2,3] and dinutuximab-beta [4], as well as the humanized anti-GD2 mAb naxitamab (previously named hu3F8) [5,6], have been approved by USA and European regulatory agencies for therapeutic treatment of high-risk NB (HR-NB). The prognosis for relapsed or refractory HR-NB, i.e., those with MYCN amplification or those diagnosed with stage 4 disease after 18 months of age, was dismal prior to the advent of immunotherapy [7,8,9].

3F8, the murine IgG3 counterpart of naxitamab, activates complement-mediated cytotoxicity (CMC) by activating complement component C1q through its Fc domain. This initiates a proteolytic cascade, forming a membrane attack complex to kill tumor cells by osmolysis [10]. 3F8 also elicits antibody-dependent cellular cytotoxicity (ADCC) when it engages Fc receptors on the surface of myeloid and natural killer cells [11]. In phase I and II trials, 3F8, in combination with granulocyte-macrophage colony-stimulating factor (GM-CSF), improved progression-free (PFS) and overall survival (OS) when used for the consolidation of first or subsequent remission when compared to historical controls [12,13]. The combination also demonstrated anti-NB activity against evaluable osteomedullary NB, though not against soft tissue disease. 

β-glucans are complex polysaccharide molecules that constitute structural cell-wall components of plants (oat, barley, seaweed), mushrooms, bacteria and yeast. Oat and barley β-glucans are primarily linear, with large regions of 1,4-β-D-glucan molecules separating shorter stretches of 1,3-β-D-glucan molecules. Fungal and yeast-derived β-glucans (BG) have more 1,6-β-D glucan branches, which are further elaborated with 1,3-β-D-glucan regions [14]. β-glucans stimulate both innate and adaptive immune responses. Being a major fungal cell-wall carbohydrate, BG acts as a pathogen-associated molecular pattern with pronounced immunomodulatory functions. It can activate several membrane-bound receptors in human leukocytes, including dectin-1 receptor, lactosylceramide and complement receptor 3 (CR3) [15]. The dectin-1 receptor is a type II transmembrane protein C-type lectin receptor expressed on monocytes, macrophages, neutrophils, dendritic cells (DCs) and a subset of T cells [16]. When dectin-1 is bound by β-glucan on macrophages, it activates the downstream signaling pathway, triggering phagocytosis, reactive oxygen species, microbial killing and cytokine production [17]. Through activation of these innate immune cells, β-glucans can act as potential vaccine adjuvants [18]. Moreover, β-glucans can enhance ADCC by CR3-dependent mechanisms. CR3 is a heterodimeric transmembrane receptor comprised of CD11b and CD18 distinct moieties expressed on neutrophils, monocytes and NK cells. iC3b binds to CD18, but this binding does not trigger the killing of cells coated with iC3b alone. However, when a second signal is provided by β-glucan, which binds to CD11b, the CR3 receptor is activated [19], resulting in cytotoxicity of iC3b-coated tumor cells. This synergism of BG with IgG monoclonal antibodies was proven for anti-GD2 3F8, anti-CD20 rituximab, anti-HER2 trastuzumab, anti-GD3 R24 and others in a broad spectrum of human xenograft models [20,21]. 

In a phase I trial, the combination of 3F8 and barley-derived β-glucan was safe, well-tolerated and achieved anti-tumor responses in patients with chemoresistant HR-NB [22]. Although MTD was not reached, two patients developed immune thrombocytopenia, possibly related to the combination. We therefore considered the use of yeast BG, which had not been tested in a formal clinical trial in children with cancer. Before incorporating BG in the overall immunotherapy approach to NB, we carried out a phase I dose-escalation trial of BG, in combination with anti-GD2 mouse 3F8, in chemoresistant NB.

## 2. Methods

### 2.1. Study Design

Patients with recurrent or refractory HR-NB were enrolled on the Memorial Sloan Kettering Cancer Center Protocol 05-073 (ClinicalTrials.gov, accessed on 13 November 2021, identifier: NCT00492167). The protocol was approved for conduct at the academic hospital Memorial Sloan Kettering Cancer Center after approval by its Institutional Review Board. Written informed consent was obtained from patients or their guardians. The primary objective was to define the toxicity of purified oral BG (Biotec Pharmacon ASA Inc., Tromso, Norway) when used in combination with intravenous (IV) 3F8. Secondary objectives included assessing disease response, survival, and immunological effects. Salient eligibility criteria included the diagnosis of HR-NB (stage 4 disease diagnosed at >18 months of age or MYCN-amplified ≥ stage 3 tumor at any age) with evaluable or measurable chemoresistant or relapsed metastatic disease. Prior anti-GD2 mAb therapy, including 3F8, was permitted. Patients with life-threatening infections or > grade 2 toxicity, according to the National Cancer Institute’s Common Toxicity Criteria for Adverse Events version 3.0 (CTCAE v3.0), were excluded. All study data and records were collected and stored on a secure institutional database. 

### 2.2. Treatment and Dose Escalation

BG was administered as 20 mg/mL aqueous solution orally once daily for 17 days (day-4 to day 12), and 3F8 was administered intravenously (IV) at 10 mg/m^2^/day for 10 days (day 1–5, 8–12). BG was dose escalated in cohorts of 3–6 patients per dose level from 10–200 mg/kg/day using a 3 + 3 design. Toxicities were graded using CTCAE v3.0 and were evaluated from day 4 of each cycle to 28 days after the first dose of 3F8. Dose-limiting toxicity (DLT) was defined as any grade 3 hematologic or non-hematologic toxicity. DLT of pain was defined as a need for >6 doses of opioids within 2 h of 3F8 administration. DLT definition excluded toxicity related to disease activity, prior therapy or co-interventions. Patients could continue therapy in the absence of progressive disease (PD), DLT or HAMA titer >1000 U/mL, and they were eligible for a total of 8 cycles, each cycle administered about 4 weeks apart.

### 2.3. Response Assessment 

Disease status was assessed using 123iodine-metaiodobenzylguanidine (MIBG), computed tomography scans, bilateral bone marrow (BM) aspirates and biopsies and graded using International Neuroblastoma Response Criteria (INRC) [23] after 2 cycles and every 2–4 cycles thereafter.

### 2.4. Correlative Studies 

HAMA titers were quantitated after every cycle as previously described [24]. Serum BG levels were determined by the Fungitell assay (Associates of Cape Cod, East Falmouth, MA, USA), which detects 1,3-β-D-Glucan pre-BG administration, and on days 1, 5, 8 and 12 of cycle 1. In order to evaluate leucocyte priming by BG, ADCC was tested on day 1 and day 11 of cycle 1 using previously described methods [25]. Briefly, LAN-1 NB cells (provided by R. Seeger, Children’s Hospital of Los Angeles) labeled with 51Cr were used as targets. Leukocytes were extracted from peripheral blood samples and studied for 3F8-independent and 3F8-dependent cell-mediated cytotoxicity among granulocytes and lymphocyte cell fractions. Target cells were opsonized with iC3b using normal human serum complement. iC3b-opsonized cells were then used to assay for CR3-dependent cytotoxicity in leukocyte fractions in the presence (iC3b ADCC) or absence of iC3b. Sargramostim (Berlex Oncology, Montville, NJ, USA) and interleukin-2 (Novartis, Basel, Switzerland) were employed in granulocyte and lymphocyte cytotoxicity assays, respectively. Plates were centrifuged at 200× *g* for 4 min at 20 °C and incubated at 37 °C for 4 h. Supernatants were harvested, and 51Cr released in the supernatant was counted. Total release was assessed by cell lysis with 10% SDS (Sigma-Aldrich, St. Louis, MO, USA), and background release was measured in the absence of cells. Lytic units were calculated [25]. Genotyping of dectin-1 for the rs3901533 single-nucleotide polymorphism (SNP) was performed using previously described methods [26].

### 2.5. Statistical Analysis 

Survival rates using time from either the first dose of 3F8 through progressive disease or death (PFS) or through death only (OS) were estimated using the Kaplan-Meier method and compared using the log-rank test. Patients alive without event were censored on the date of last follow up. Cox proportional hazard models were used to assess prognostic values of continuous variables. The Mann-Whitney Wilcoxon test (2 groups) was used for association studies between the dose level of BG and correlative studies (iC3b ADCC, ADCC, Dectin polymorphism, HAMA, BG levels in blood), and Kruskal-Wallis tests (3 groups) were used for association studies between dose level of BG and response. Fisher’s exact test was used for correlation between two categorical variables.

## 3. Results

### 3.1. Patients

Forty-four patients with relapsed/refractory HR-NB (29 male; 15 female) with a median age at enrollment of 6.2 (1–26.3 years) were treated between 2006 and 2008. Patient characteristics are shown in Table 1. At study enrollment, 11 (25%) patients had disease refractory to initial chemotherapy (termed primary refractory), 21 (48%) had NB that had no response or incomplete response to second-line chemotherapy after relapse (secondary refractory group) and 12 (27%) had progressive disease (PD) immediately prior to enrollment. All patients had received dose-intensive induction chemotherapy, and 25 (57%) had undergone autologous stem-cell transplant. Thirteen patients had received prior anti-GD2 mAb: 9 (20%) with 3F8 and 4 (9%) with dinutuximab. Overall, 31 patients (70%) had one or more relapses prior to enrollment. A total of 141 cycles of yeast β-glucan and 3F8 were administered, with patients receiving 1 (*n* = 12), 2 (*n* = 9), 3 (*n* = 5) or ≥4 (*n*= 18) cycles (Supplementary Table S1).

### 3.2. Toxicities

All 44 patients were evaluable for toxicity (Table 2). In general, oral BG was well-tolerated. One dose-limiting toxicity likely related to BG (transient self-limited grade 3 elevation in hepatic enzymes) occurred at 120 mg/kg/dose and developed 5 days after starting yeast BG but before 3F8 initiation. An additional three patients were therefore treated at this dose level prior to escalation (Supplementary Table S1). Maximum tolerated dose (MTD) for BG was not reached, despite escalation to 200 mg/kg/dose. There was no other grade >2 therapy-related toxicity, and all other toxicities were expected and related to 3F8. The latter included pain, allergic reactions, hypotension and hypertension. No related delayed or long-term toxicities were detected among the 27 patients after 2 years or among the 16 survivors after 5 years. Reasons for withdrawal among the 40 patients who did not complete the planned number of cycles were DLT in one patient (2%), two persistently elevated human anti-mouse antibody (HAMA) responses in six patients (15%), PD in 23 patients (58%) and patient/physician preference in 10 patients (25%).

### 3.3. Disease Responses and Survival

**Figure 1:**
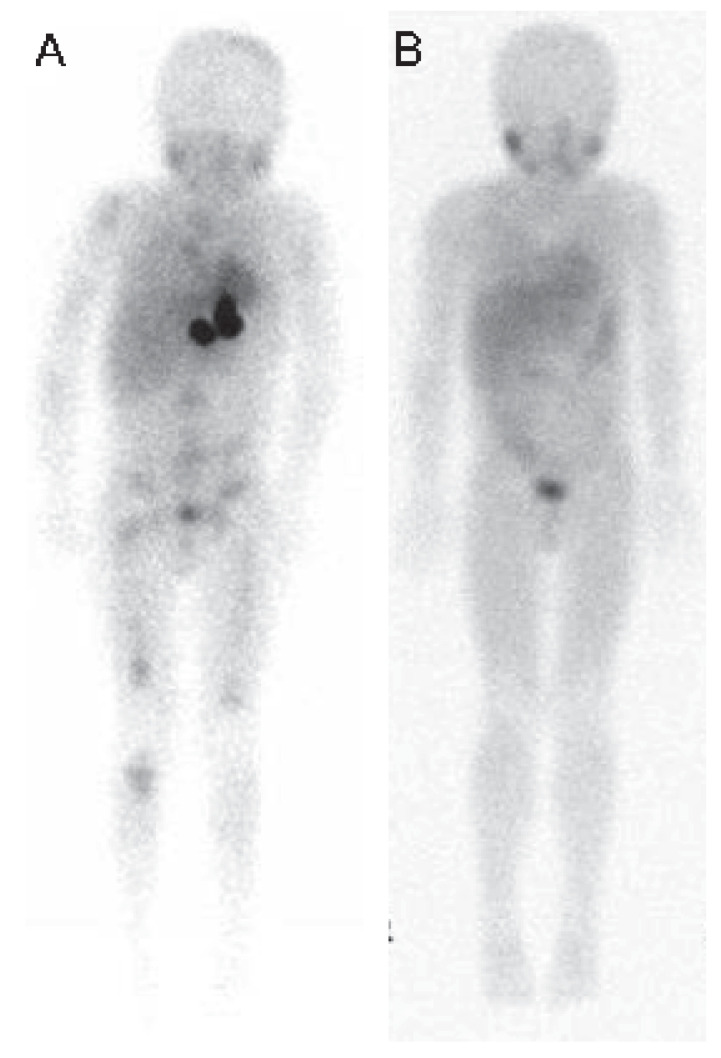
Clinical response of a neuroblastoma patient to 3F8 plus yeast beta-glucan. ^123^MIBG scans before the start of 3F8 plus yeast β-glucan (**A**) and after (**B**) 4 cycles of 3F8 plus yeast β-glucan. The skeletal uptake of ^123^MIBG (seen in skull, appendicular skeleton, pelvis and vertebrae) resolved after treatment with 3F8 plus yeast BG.

Responses after two cycles and best response on protocol are summarized in Supplementary Table S2. A total of 41 out of 44 patients were evaluable by INRC. Best responses were complete response in 2 (5%), partial response in 3 (7%), stable disease in 23 (56%) and PD in 13 (32%) patients. Responses were noted in osteomedullary disease, but none of the 21 patients with evaluable soft tissue disease demonstrated response. One patient had a near complete remission of skeletal MIBG uptake (Figure 1). An additional 4/28 patients had reduction in osteomedullary scores. Among evaluable patients with BM involvement, 4/6 (67%) achieved a complete response in that site. There was no evidence that responders and non-responders at the end of treatment received a different dose of BG (Kruskal-Wallis rank sum test; *p* = 0.81). At 2 years, the PFS rate was 28%, and the OS rate was 61%; at 5 years, they were 19% and 36%, respectively.

### 3.4. Correlative Studies

**Figure 2:**
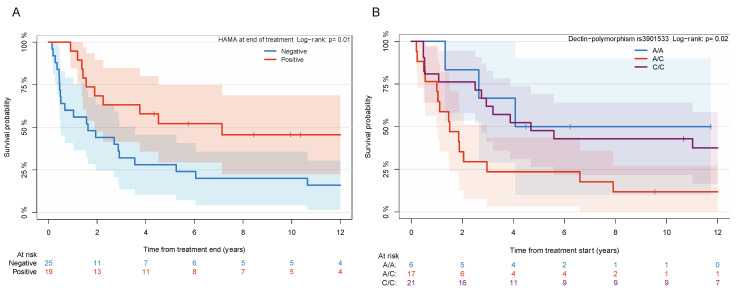
Correlative studies and overall survival. (**A**) Overall survival post-treatment by HAMA status during treatment. (**B**) Overall survival by Dectin-polymorphism rs3901533 genotype.

Univariable models identified significant prognostic factors for both PFS and OS. HA-MA-positivity developed in 19 (43%) patients during treatment at a median of 2 (range 0.8–20.4) months from start of 3F8 and 6 (14%) patients with persistently elevated HAMA did not continue treatment. There was no significant difference between developing HA-MA and history of prior mAb therapy; HAMA developed in 2/9 (22%) patients who had received prior anti-GD2 mAbs and in 17/35 (49%) of anti-GD2 mAb-naïve patients (*p* = 0.26 by Fisher’s Exact test). Furthermore, there was no evidence that patients with HA-MA-positivity had received a different BG dose level than those with negative titers (*p* = 0.36 by Wilcoxon rank-sum test). Patients that developed HAMA at the end of treatment had a longer OS than those who did not (log-rank *p* = 0.01; Figure 2A).

Dectin-1 SNP was significantly associated with OS (*p* = 0.02) but not PFS (*p* = 0.07). Patients with dectin-1 rs3901533 A/C (17 patients; 39%) had a shorter OS than those with Dectin-1 SNP rs3901533 A/A (6 patients; 14%) or C/C (21 patients, 48%) (log-rank *p* = 0.02; Figure 2B). Patients with the SNP rs3901533 A/A polymorphism developed HAMA more frequently than those with other phenotypes. HAMA developed in 100% of patients in the A/A group, versus 35% in the A/C group and 33% in the C/C group (*p* = 0.01).

In cytotoxicity assays, the administration of 3F8 plus BG did not significantly affect in vitro anti-NB responses. Furthermore, there was no correlation of BG dose with these parameters (iC3b-ADCC, *p* = 0.91) or absence of iC3b (ADCC, *p* = 0.25) (Mann-Whitney-Wilcoxon test). (1,3) β-glucan was detected in the serum of all (28/28) patients tested with the Fungitell assay. Furthermore, there was a moderate correlation between BG dose and the level of BG in the serum of the 28 patients with measured BG levels (Spearman’s coefficient is 0.624, Supplementary Figure S1). There was no evidence of a difference in serum BG levels for patients with and without HAMA at the end of treatment (Wilcoxon rank sum test, *p* = 0.12).

## 4. Discussion

This phase I trial in patients with resistant HR-NB assessed the toxicity of escalating dosages of the immunological adjuvant BG when used in combination with fixed doses of anti-GD2 antibody 3F8. We demonstrated that the combination of BG and 3F8 is safe, even with BG doses of up to 200 mg/kg/dose. There were no major adverse events related to BG, and the known adverse events related to 3F8 (pain, allergic reactions) were not exacerbated. The sole DLT was self-limited. MTD of BG was not reached, and the study was completed at the planned dose of 200 mg/kg/day. While the post-3F8/BG treatment was variable and patient selection bias was inevitable in single-center trials, 27 patients survived past 2 years, and 16 patients survived past 5 years—61% and 36%, respectively, an improvement over the dismal outcome reported in recent summaries [7,8,9].

Our prior experience with patients receiving 3F8 as a single agent or an agent in combination with GM-CSF suggested that the development of HAMA was associated with an improved OS. We made a similar observation in this study. The proportion of patients developing HAMA was higher in this study when compared to those treated with barley-derived BG [22] or those treated with 3F8 + GM-CSF (43% vs. 25%) in other studies. Moreover, patients receiving 3F8 + BG appeared to develop HAMA relatively quickly in this study, with a median time to develop HAMA of 2 months from initiation of 3F8. In comparison, median time to develop HAMA in patients receiving 3F8 + GM-CSF was 9 months. While early-onset neutralizing HAMA might have a negative impact by limiting the amount of murine mAb therapy, previous studies have implicated the anti-idiotype network as a vaccination effect whereby ant-GD2 antibodies could be induced to prevent tumor progression [27]. Furthermore, the favorable prognostic value of HAMA on overall survival was demonstrated among relapsed patients salvaged with 3F8 + GMCSF [13] and in first-remission patients treated with 3F8 + GMCSF [12].

The dectin-1 receptor appears to play a critical role in the function of BG. Dectin-1 has a broad role in defense against pathogens and as an effective targeting strategy for cancer treatment [28]. The rs3901533 polymorphism might negatively impact the function of the dectin-1 receptor, and it has been associated with an increased susceptibility to and severity of invasive fungal infections [29]. The statistical association of the wild-type allele rs3901533 A/A with higher HAMA response is consistent with its adjuvant effect on the immune system in stimulating the human anti-mouse HAMA response, as well as the accelerated pace of this response. The potential of BG as a vaccine adjuvant, previously shown in mice, was tested in two subsequent vaccine trials, a phase I [30] and a phase II [26]. In the GD2/GD3 vaccine plus BG phase II trial, there was an association between anti-GD2 IgG1 titers and SNP rs2901533, again confirming the role of the dectin-1 receptor for BG-mediated vaccine adjuvant effect. In that case, no major toxicities were encountered, and overall survival was near 90% [26].

It is noteworthy that when 3F8 was combined with barley-derived (1,3) (1,4) β-glucan (clinicaltrials.gov, accessed on 13 November 2021, NCT00037011), the MTD for barley β-glucan was, similarly, not reached. However, there were two patients that developed grade 4 thrombocytopenia after the first cycle of therapy at a dose of 20 mg/kg/day and 40 mg/kg/day, respectively. This phenomenon was thought to be autoimmune in nature, possibly due to BG-mediated activation of CR3 [22]. Although the toxicity profile of BG, even at doses as high as 200 mg/kg/dose, was favorable, the volume of BG required to be ingested has, on occasions, proved to be onerous for young children. Hence, the dose of 40 mg/kg was adopted for all subsequent studies.

As expected for a phase I trial in a heavily pre-treated group of patients with chemoresistant HR-NB, including >70% patients with relapsed disease and many with multiple relapses, the overall response rate was relatively modest, similar to that reported with 3F8 in the past. Additionally, as previously observed for anti-GD2 antibody therapy without additional chemotherapy, we did not see responses of soft tissue disease. Moreover, whether the addition of BG to humanized 3F8, which has enhanced ADCC properties compared to 3F8, will be beneficial remains to be investigated. Nevertheless, its lack of significant toxicity, its potential to bridge innate (CR3) and adaptive (dectin-1) immune mechanisms and its vaccination adjuvant effect provide a scientific basis for incorporating BG into the anti-GD2 immunotherapy strategy for NB.

## 5. Conclusions

Yeast β-glucan was well tolerated and lacked major toxicity. In the presence of 3F8, activity against resistant osteomedullary neuroblastoma was noted. The role of yeast β-glucan in enhancing effects of anti-cancer immunotherapy is now being tested in anti-GD2 vaccine trials.

## 6. Patents

A patent on yeast β-glucan use in immunotherapy was filed by MSK and was licensed to Biotec Pharmacon.

## Figures and Tables

**Table 1:** Characteristics of the patients at baseline.

Characteristic	*N* = 44
Median age at diagnosis (years)	4.2 [range: 0.2–25.1]
Median age at start of therapy (years)	6.2 [range: 1.0–26.3]
*MYCN*	
Amplified Non-amplified	17 (39%) 27 (61%)
*Relapse status prior to study entry*	
Primary Refractory	11 (25%)
Progressive Disease	12 (27%)
Secondary Refractory	21 (48%)
Prior 3F8 therapy	9 (20%)
Prior ASCT	25 (57%)
Median number of prior relapses	1 [0–3]

Abbreviations: ASCT, autologous stem cell transplant.

**Table 2:** Treatment-related toxicities.

Toxicity	Grade 1	Grade 2	Grade 3	Total (*N* = 44)
Pain	2	42	0	44
Nausea	4	0	0	4
Vomiting	5	5	0	10
Urticaria	0	18	0	18
Pruritus	1	19	0	20
Flushing	3	7	0	10
Cough	2	0	0	2
Edema	1	3	0	4
Fatigue	0	2	0	2
Fever	1	0	0	1
Elevated AST	20	4	1*	25
Elevated ALT	16	7	1*	25
Hypertension	3	0	0	3
Anxiety/agitation	4	0	0	4

Only toxicity related to BG (recorded as DLT in one patient). All other toxicities were expected and related to 3F8. Abbreviations: AST, aspartate aminotransferase; ALT, alanine aminotransferase.

## Data Availability

Requests for deidentified individual participant data can be made beginning 12 months after publication and for up to 36 months post publication. Deidentified individual participant data reported in the manuscript will be shared under the terms of a Data Use Agreement and may only be used for approved proposals. Requests may be made to: crdatashare@mskcc.org.

## Supplementary Materials

The following are available online at https://www.mdpi.com/article/10.3390/cancers13246265/s1, Figure S1: BG level by dose, Table S1: Numbers of cycles administered, Table S2: Responses by INRC.
