# Supplementary material for: Phase I Trial of Oral Yeast-Derived β-Glucan to Enhance Anti-GD2 Immunotherapy of Resistant High-Risk Neuroblastoma
Source: Cancers (Basel). 2021 Dec 14;13(24):6265. doi: 10.3390/cancers13246265 (PMC8699451; doi:10.3390/cancers13246265)
Supplement: Supplementary file 1 [file cancers-13-06265-s001.zip › cancers-1486369-supplementary.pdf]

Table S1. Number of cycles administered

BG Dose* (mg/kg)	Overall N=44	Number of cycles administered N=141
**10**	3	12
**20**	3	13
**40**	3	7
**80**	6	17
**100**	3	8
**120**	6	17
**140**	6	17
**160**	5	17
**180**	3	10
**200**	6	23

Abbreviations: BG, β-glucan.

* The initial planned maximum dose was 120mg/kg. However, since there were no significant adverse events encountered, additional dose levels were added after IRB approval. At a dose level of 160mg/kg, one patient did not complete the entire course of BG, and one patient was treated on a compassionate basis.

Table S2. Responses by INRC

	**CR**	**PR**	**SD**	**PD**
Response after 2 cycles (n=41)*	0 (0%)	4(10%)	24 (59%)	13(32%)
Best Response on protocol (n=41)*	2 (5%)	3 (7%)	21 (48%)	15 (34%)

Abbreviations: CR, Complete response; INRC, International Neuroblastoma Response Criteria; PD, progressive disease; PR, Partial response; SD, Stable Disease
*Three patients were non-evaluable.

Figure S1. BG level by dose

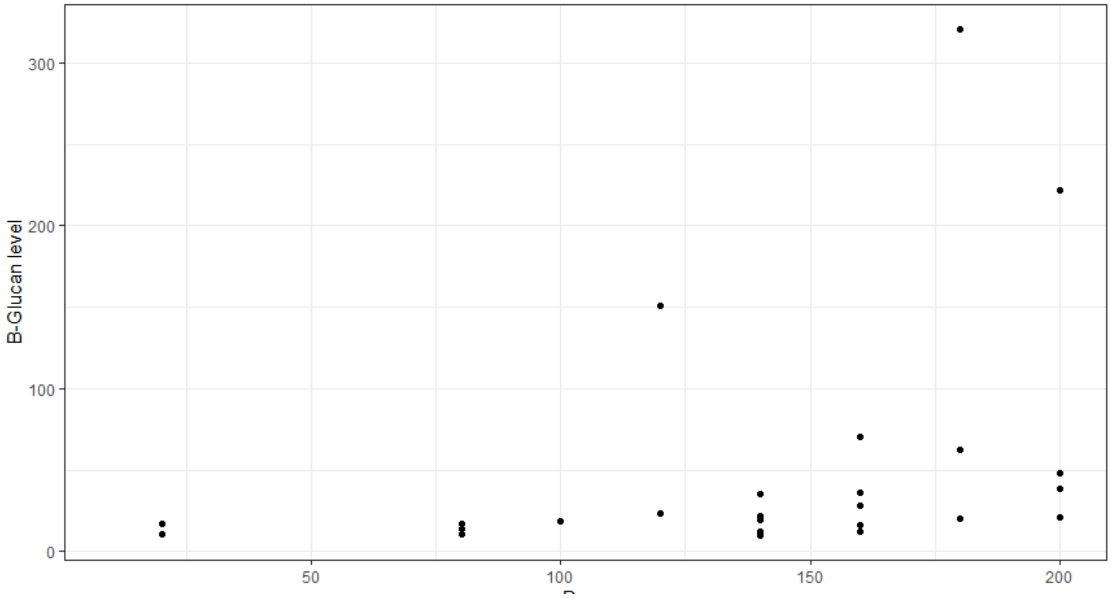

BG measured as (1,3) β-glucan
